# Optical coherence tomography findings of falciform retinal detachment complicated with persistent fetal vasculature

**DOI:** 10.1186/s12886-018-0893-0

**Published:** 2018-08-29

**Authors:** Daisaku Kimura, Masanori Fukumoto, Takaki Sato, Ryohsuke Kohmoto, Takatoshi Kobayashi, Teruyo Kida, Tsunehiko Ikeda

**Affiliations:** 1 Department of Ophthalmology, Osaka Medical College, 2-7 Daigaku-machi, Takatsuki City, Osaka, 569-8686 Japan; 2 Department of Ophthalmology, Takatsuki Red Cross Hospital, Takatsuki-City, Osaka, Japan

**Keywords:** Falciform retinal detachment (FRD), Visual function, Optical coherence tomography (OCT), Persistent fetal vasculature (PFV)

## Abstract

**Background:**

Falciform retinal detachment (FRD) usually causes pronounced retinal wrinkles, and the prognosis of visual function is poor. In this present study, we report a rare case of FRD in which optical coherence tomography (OCT) findings revealed a relatively good visual function.

**Case presentation:**

This study involved a 22-year-old female who had previously been diagnosed with FRD at 2 years of age, and who presented with microphthalmus in both eyes with pronounced retinal folds from the optic disc to the inferior-temporal side. Based on the clinical findings, we diagnosed it as persistent fetal vasculature (PFV). We found the visual function in her left eye to be relatively poor, yet from 6 to 22 years of age, the corrected visual acuity in that eye remained at 0.08. Although a nystagmus was present, Goldman perimetry showed a relatively wider visual field than expected. Optical coherence tomography (OCT) findings revealed that the retinal layer structure near the FRD was relatively well maintained, except for the temporal peripheral region.

**Conclusions:**

Our findings reveal that OCT examination can be considered useful for predicting the visual function in cases of FRD.

## Background

Clinical findings of persistent fetal vasculature (PFV) reportedly include cataracts, glaucoma, shallow anterior chamber, the extension of ciliary processes, retinochoroidal degeneration, a dragged retina, falciform retinal detachment (FRD), leukocoria, and corneal opacity, etc., which can be classified into the anterior segment type and posterior segment type [1–3]. Although PFV is thought to primarily occur in one eye, it reportedly can occur bilaterally in approximately 10% of the cases [2]. FRD is known to occur in cases of posterior-segment-type PFV. The present case involved a 22-year-old female who was delivered at full-term of pregnancy and her family had no previous history of eye-related disease, so we theorize that the FRD was most likely caused by posterior-segment-type PFV.

FRD, also referred to as “congenital falciform fold”, is a special-traction retinal detachment that reportedly forms as a result of strong contractile force and hyperextensibility of the retina, in which fibrovascular tissue is formed in the peripheral vitreoretinal surface during the perinatal period or infancy [4]. Usually, retinal folds are formed toward the inferior-temporal side. Diseases that cause FRD include familial exudative vitreoretinopathy (FEVR), PFV, retinopathy of prematurity, incontinentia pigmenti, and ocular toxocariasis [4, 5]. Most patients with FRD usually experience poor visual function, as the macular portion becomes entangled in the retinal folds. In this present study, we report a case of FRD in which relatively good visual acuity and visual field were retained in comparison to findings of the fundus, and analyze its correlation with the optical coherence tomography (OCT) findings and visual function.

## Case presentation

This present case involved a 22-year-old female who became aware of leukocoria in both eyes. Upon examination at another eye clinic, a vitreous strand was detected in her left eye, with a suspected diagnosis of PFV, and she was referred to the Department of Ophthalmology at Osaka Medical College Hospital, Takatsuki-City, Japan for a more detailed diagnosis and subsequent treatment.

The patient had previously been diagnosed with strabismus when she was 2 years of age. She was delivered at full term, with a birth weight of 3320 g, and she had no history of oxygen administration. We did not perform genetic investigation (i.e., sequencing) on the patient in order to diagnose PFV. In addition, her relatives had no previous history of visual impairment.

**Fig. 1:**
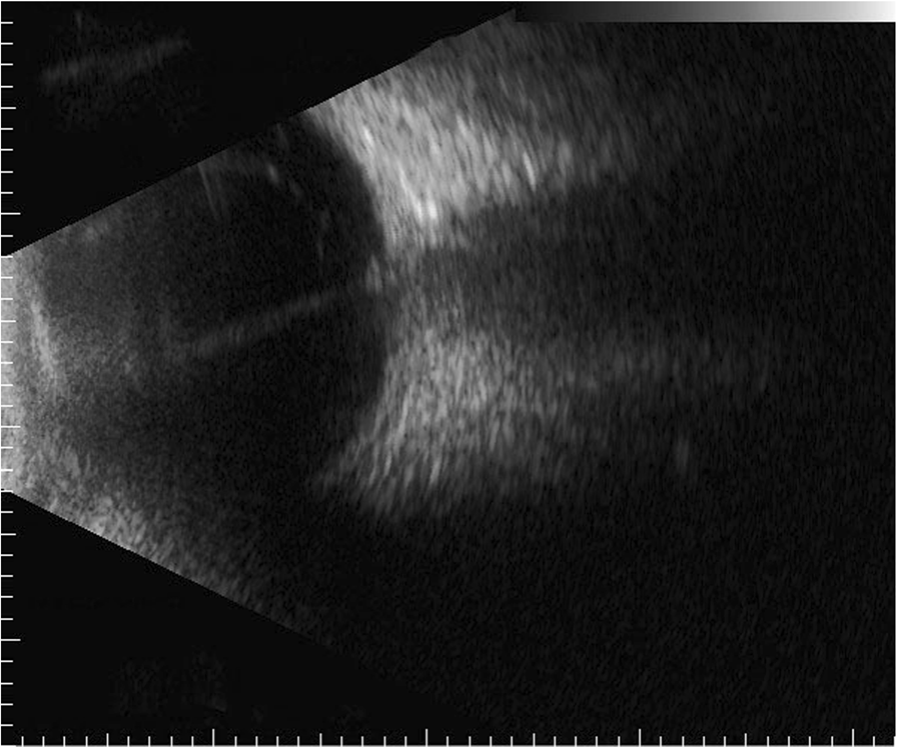
B-scan ultrasonography image of the right eye of the 22-year-old female patient with falciform retinal detachment (FRD). Total retinal detachment can be seen

**Fig. 2:**
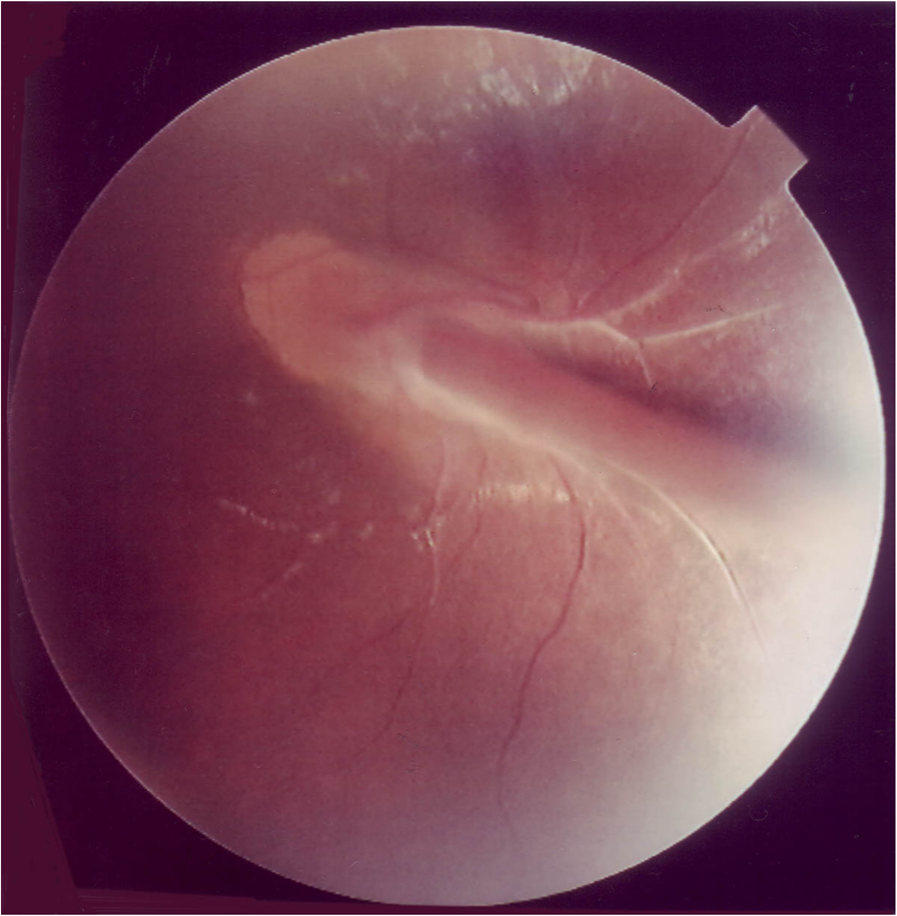
Fundus images of a 22-year-old female with falciform retinal detachment (FRD) that were obtained when the patient was 6 years of age. The fundus exhibited FRD from the optic disc to the inferior-temporal side

At initial visit, the clinical findings of a slit-lamp examination revealed a shallow anterior chamber in both eyes. In her right eye and left eye, the diameter of the cornea was 8 mm and 9 mm and the axial length was 15 mm and 19 mm, respectively, and microphthalmus was observed in both eyes. In her right eye, the fundus was not visible due to a cataract, and ultrasonic B-mode examination revealed total retinal detachment (Fig. 1). A magnetic resonance imaging scan of the patient’s head revealed no calcification in the right eye and no abnormalities in her brain. In the left eye, retrolental fibrovascular proliferation was found around the temporal side. The fundus exhibited FRD from the optic disc to the inferior-temporal side (Fig. 2). Most of the peripapillary retinal vessels were involved in the retinal folds, and a part of the nasal retina covered-over the optic disc. In the periphery of the fundus, retinal avascular area was observed over the entire circumference, and pigmentation was also observed in a wide range on the temporal side. An oscillating nystagmus was observed in both eyes, and was found to be prominent in the left gaze and less conspicuous in the right gaze when her face was turned to the left.

**Fig. 3:**
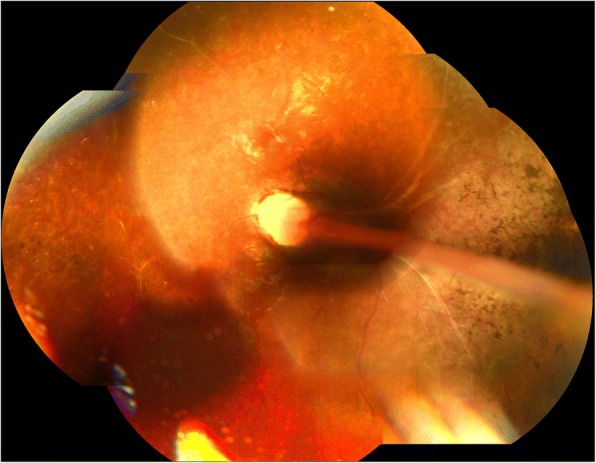
Fundus images obtained when the patient was 22 years of age. No significant change in the fundus findings can be seen in comparison to those obtained when she was 6 years of age (Fig. 2)

**Fig. 4:**
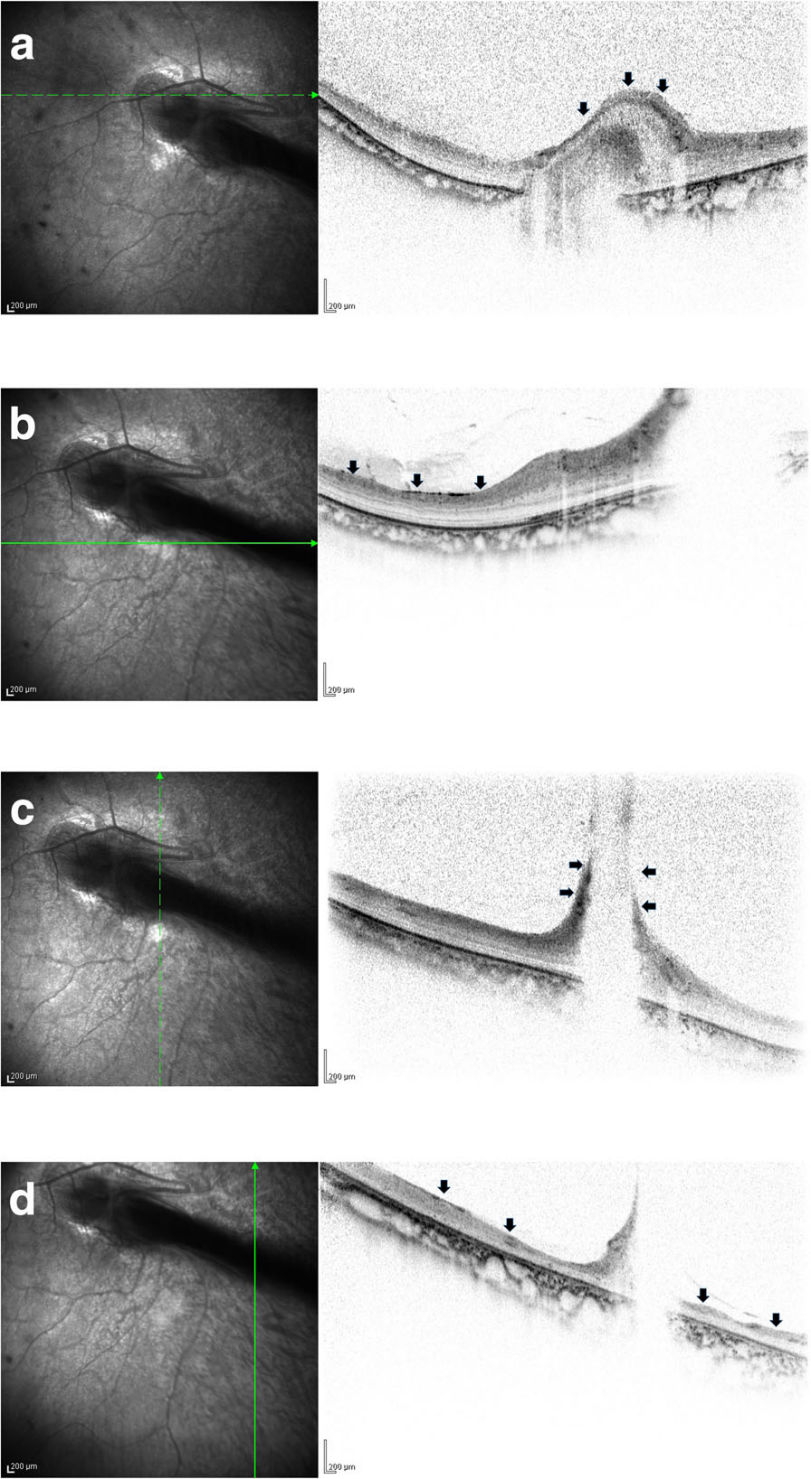
Optical coherence tomography (OCT) images of the patient’s left eye that were obtained when she was 22 years of age. **a** OCT image showing bundle shading at the optic disc combined with the finding that the nasal retina was overlaid on the optic disc (arrows). **b** OCT image showing that the layer structure of the surrounding retina was well preserved (arrows). **c** OCT image of the temporal side of the optic disc showing elevated stalk of the fold protruding into the vitreous at the site of the FRD (arrows), yet the upper and lower retinal layered structures were relatively well retained. **d** OCT image of the temporal peripheral side, showing that the retina was remarkable thinned, the layered structure was unclear and that the ellipsoid zone could not be clearly identified (arrows)

During the clinical course, cataract and corneal opacification progressed, ultimately becoming phthisis bulbi in her right eye. On the other hand, from the age of 6 to 22 years, her left eye retained a corrected visual acuity of 0.08, and no significant change of the fundus was observed during that 16-year period (Fig. 3). OCT images obtained when she was 22 years of age revealed bundle shading at the optic disc, combined with the finding that the nasal retina was overlaid on the optic disc (Fig. 4a). However, the layer structure of the surrounding retina was well preserved (Fig. 4b). On the temporal side of the optic disc, the elevated stalk of the fold protruding into the vitreous was observed at the site of the FRD, yet the upper and lower retinal layered structures were relatively well retained (Fig. 4c). However, on the temporal peripheral side, the retina was remarkable thinned, the layered structure was unclear, and the ellipsoid zone could not be clearly identified (Fig. 4d).

**Fig. 5:**
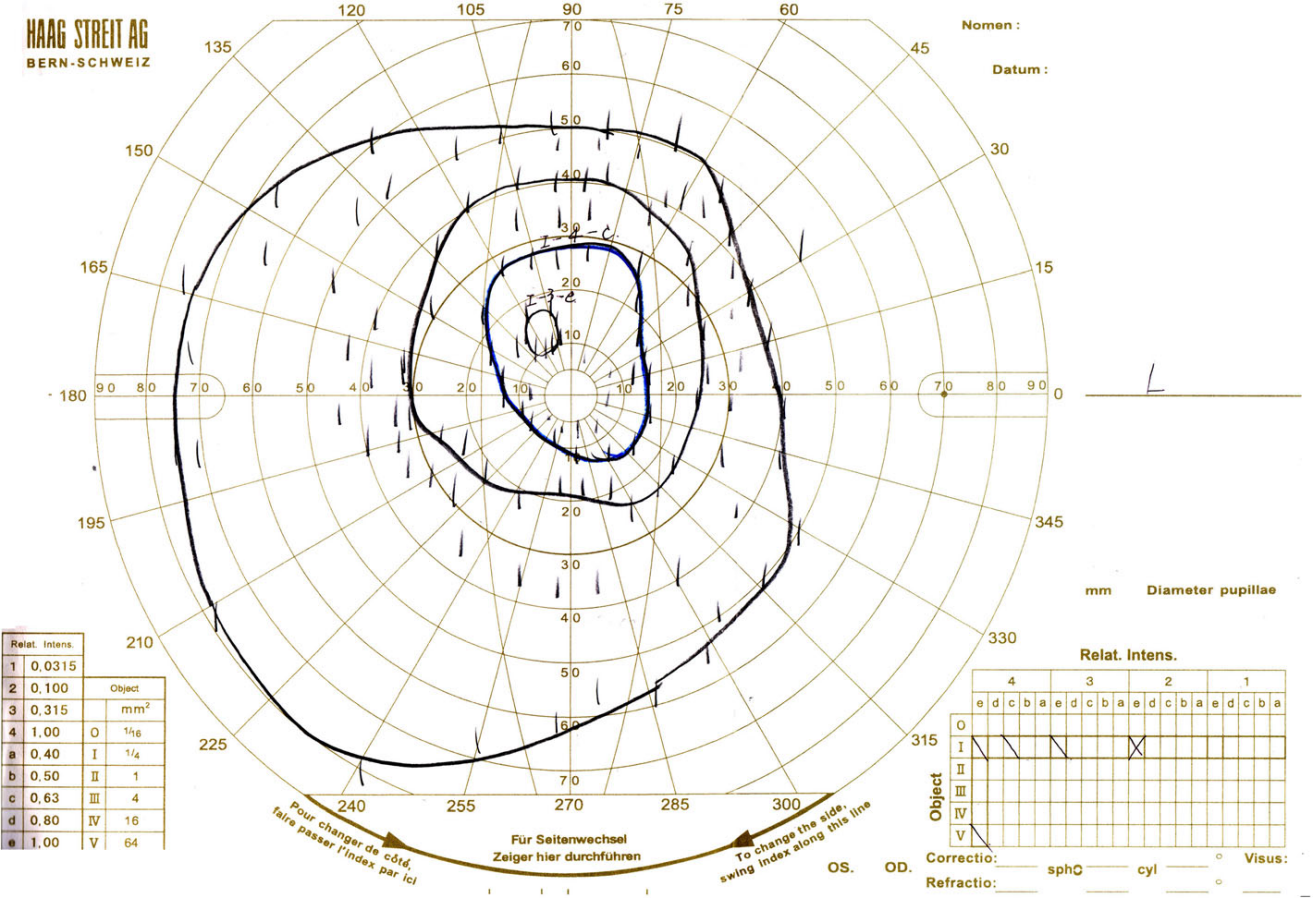
Chart showing the Goldman kinetic visual-field examination findings that were obtained when the patient was 22 years of age. Goldman perimetry with an isopter of V-4 showed a relatively wider visual field than we had expected

Goldman kinetic visual field examination findings, with an isopter of V^− 4^, obtained when the patient was 22 years of age exhibited 50-degrees upwards, 40-degrees to the nasal side, 60-degrees downward, 75-degrees to the temporal side, and 80-degrees to the inferior-temporal side (Fig. 5). The patient is currently undergoing yearly follow-up observations (i.e., once per year).

## Discussion

It should be noted that most FRD cases have very poor visual function, as the macular region is involved in the retinal folds. In this present case, the corrected visual acuity was 0.08, and a favorable visual field was maintained in comparison to the fundus findings. A primary reason for this could be that the retinal folds were slightly thin, the area of the retina surrounding the folds was relatively narrow, and the OCT findings indicated that the layer structure of the retina near the folds was relatively well preserved.

Reports of investigating the retinal condition of FRD by OCT are mainly found in cases of FEVR [5–8]. Lee et al. reported that the retina of FEVR could be observed using a hand-held OCT device, and that the vitreoretinal traction gave rise to thickening of the retinal nerve fiber layer and retinal elevation at the optic disc [6]. Yonekawa et al. reported performed OCT in 74 eyes of 41 FEVR patients, and stated that the decreased retinal thickness and the unclear ellipsoid zone were the causes of poor visual function [7]. In that study, the authors found that in addition to observation of the fundus, OCT to be extremely useful for predicting visual function. Katagiri et al. previously reported using ‘swept source OCT’ (SS-OCT) for the evaluation of 29 eyes in 18 FEVR patients complicated with FRD [8]. In that study, the authors’ results indicated three characteristic SS-OCT findings: 1) the presence of a long, tapering sensory retina from the lesion side to the optic disc, 2) slippage of the sensory retina from the optic disc to the lesion side, and 3) movement of the sensory retina from the contralateral side of the lesion to the optic disc. The authors also described that the changes in the sensory retina in the lesion area were classified into 2 groups: 1) the presence of only a long, tapering sensory retina, and 2) both a long, tapering sensory retina and slippage of the sensory retina.

In the present case, the nasal retina was found to cover the optic disc, and the OCT results were found to be consistent with that finding. In addition, although OCT revealed a large single bundle of shadows on the optic disc, this finding is similar to the severe cases of FEVR reported by Katagiri et al. In the area of the FRD on the peripheral side, it was recognized as a shadow protruding into the vitreous cavity, which is similar to the OCT findings in the case reported by Yonekawa et al. However, in those previously reported cases, as well as in our present case, the retinal layer structure was relatively well preserved around the FRD. It has been theorized that there is a correlation, at least to some extent, that visual acuity and visual field are relatively better than what was predicted by the ophthalmoscopic findings in the presented case. In addition, it was confirmed by the Goldman dynamic visual field examination that the retina on the temporal side was thinner compared to the nasal side, that the retinal layer structure was somewhat obscure, and that the ellipsoid zone could not be clearly confirmed in the temporal side. This is consistent with a somewhat narrowing of the visual field in the direction that coincides with the temporal retina.

In closing, compared with the usual cases, OCT was somewhat difficult to perform in this present case due to the nystagmus, however, and as reported by Yonekawa et al., it is thought that OCT can be a useful examination to predict the visual function in cases with FRD.

## Conclusions

These findings revealed that OCT examination can be considered useful for predicting the visual function in cases of FRD.

## Abbreviations

FEVR: familial exudative vitreoretinopathy
FRD: falciform retinal detachment
OCT: optical coherence tomography
PFV: persistent fetal vasculature
SS-OCT: swept source OCT
